# Mechanical fibrinogen-depletion supports heparin-free mesenchymal stem cell propagation in human platelet lysate

**DOI:** 10.1186/s12967-015-0717-4

**Published:** 2015-11-10

**Authors:** Sandra Laner-Plamberger, Thomas Lener, Doris Schmid, Doris A. Streif, Tina Salzer, Michaela Öller, Cornelia Hauser-Kronberger, Thorsten Fischer, Volker R. Jacobs, Katharina Schallmoser, Mario Gimona, Eva Rohde

**Affiliations:** Spinal Cord Injury and Tissue Regeneration Center Salzburg, Paracelsus Medical University, Salzburg, Austria; Department of Blood Group Serology and Transfusion Medicine, Spinal Cord Injury and Tissue Regeneration Center Salzburg, Paracelsus Medical University, Lindhofstrasse 20-22, 5020 Salzburg, Austria; Department of Pathology, Paracelsus Medical University, Salzburg, Austria; Department of Gynecology and Obstetrics, Paracelsus Medical University, Salzburg, Austria

**Keywords:** Fibrinogen, Heparin, Mesenchymal stem cells, Pooled human platelet lysate

## Abstract

**Background:**

Pooled human platelet lysate (pHPL) is an efficient alternative to xenogenic supplements for ex vivo expansion of mesenchymal stem cells (MSCs) in clinical studies. Currently, porcine heparin is used in pHPL-supplemented medium to prevent clotting due to plasmatic coagulation factors. We therefore searched for an efficient and reproducible medium preparation method that avoids clot formation while omitting animal-derived heparin.

**Methods:**

We established a protocol to deplete fibrinogen by clotting of pHPL in medium, subsequent mechanical hydrogel disruption and removal of the fibrin pellet. After primary culture, bone-marrow and umbilical cord derived MSCs were tested for surface markers by flow cytometry and for trilineage differentiation capacity. Proliferation and clonogenicity were analyzed for three passages.

**Results:**

The proposed clotting procedure reduced fibrinogen more than 1000-fold, while a volume recovery of 99.5 % was obtained. All MSC types were propagated in standard and fibrinogen-depleted medium. Flow cytometric phenotype profiles and adipogenic, osteogenic and chondrogenic differentiation potential in vitro were independent of MSC-source or medium type. Enhanced proliferation of MSCs was observed in the absence of fibrinogen but presence of heparin compared to standard medium. Interestingly, this proliferative response to heparin was not detected after an initial contact with fibrinogen during the isolation procedure.

**Conclusions:**

Here, we present an efficient, reproducible and economical method in compliance to good manufacturing practice for the preparation of MSC media avoiding xenogenic components and suitable for clinical studies.

**Electronic supplementary material:**

The online version of this article (doi:10.1186/s12967-015-0717-4) contains supplementary material, which is available to authorized users.

## Background

The isolation and ex vivo expansion of mesenchymal stem cells (MSCs) is a prerequisite for clinical evaluation regarding their promotion of various therapeutic effects such as tissue regeneration, neuroprotection or immunomodulation [1, 2]. Cell culture media used for in vitro expansion frequently contain fetal bovine serum (FBS) as a source of growth factors. Even though widely applied, major concerns regarding the use of animal serum have emerged: Bovine serum harbors the risk of xenogenic immune reactions. Human MSCs cultured in FBS were shown to generate immune responses in patients receiving MSC-based therapies [3–5]. This might be due to bovine proteins, that were shown to be internalized either directly into the cells [6] or transferred by bovine extracellular vesicles [7]. Furthermore, pathogens such as viruses, mycoplasma and prions can be transmitted by animal sera [8–11]. In addition, bovine sera vary from batch to batch with regard to their effectiveness to support cell proliferation [12, 13]. Because of these concerns, the use of FBS in human cell culture applied for stem cell therapies is not recommended to date [14, 15]. Thus, a growing demand for human alternatives such as pooled human platelet lysate (pHPL) has emerged for clinical trials investigating MSCs [16]. pHPL contains abundant growth factors and cytokines to efficiently boost MSC proliferation in vitro [17, 18]. Further, pHPL components include plasmatic coagulation factors such as fibrinogen and various platelet-derived factors [19, 20]. As a result of physiological coagulation, pHPL added to calcium-containing growth media rapidly form viscoelastic fibrin gels. To prevent clot formation in cell culture, heparin, a highly sulfated glycosaminoglycan [21], is commonly added. It has been shown that the concentration and quality of heparin, usually of porcine origin, and the added preservatives therein are critical for cell culture with respect to proliferation, colony forming and ex vivo differentiation capacity of mesenchymal stem cells, but also various other cell types [22–27]. Data further indicate that heparin disrupts the CXCR4/SDF-1 signaling axis and may interfere with migration and homing capacity of BM-derived mononuclear cells [28]. Therefore it has been suggested that heparin should be supplied at lowest possible concentrations in media containing pHPL to prevent gel formation [26]. It may be not only beneficial to avoid heparin, but also to deplete fibrinogen which is a known pro-inflammatory component of pHPL [29]. A recent study demonstrated that fibrinogen increases the adhesion of peripheral blood human natural killer cells, which in turn stimulate human BM-MSC invasion and may not only induce tissue repair but also an exacerbated inflammatory response [29]. Recent data suggest that fibrinogen may negatively affect the immune modulating capacity of MSCs [30]. Considering numerous clinical trials for the treatment of immune disorders, fibrinogen-depletion in pHPL-containing growth media is potentially useful. To date procedures to reduce fibrinogen and to avoid heparin are based on the addition of CaCl_2_ to undiluted pHPL to antagonize citrate effects and to induce the coagulation cascade, thus producing serum-converted platelet lysate [30, 31].

In our study, we searched for alternative methods to deplete fibrinogen in pHPL-supplemented media while preventing the use of any additional reagents such as CaCl_2_ or porcine heparin. Fibrin polymers rapidly assemble by a modified polycondensation reaction from fibrinogen resulting in three-dimensional networks with large elastic moduli [32, 33]. Owing to the hydration and the physical properties of viscoelastic fibrin polymer gels, the networks are sensitive to strain and excessive strain burden causes the collapse and aggregation of the fibrin fibers. We have applied a strategy based on this fibrin gel physics to remove fibrinogen from pHPL-based cell culture media without additional requirement for further chemical support. Following our protocol, the resulting complete growth medium remains clear and no further gel or fiber formation is observed. Here we show that heparin free and fibrinogen depleted media are equally efficient to standard pHPL media containing heparin for the cultivation of human MSCs. The here described method is the first to gain entirely humanized cell culture media with high medium recovery rates after induced clotting and low remaining fibrinogen amounts. Thus, it facilitates a standardized and GMP-grade generation of medium for future clinical studies.

## Methods

### Medium preparation, fibrinogen depletion and quantification

**Fig. 1:**
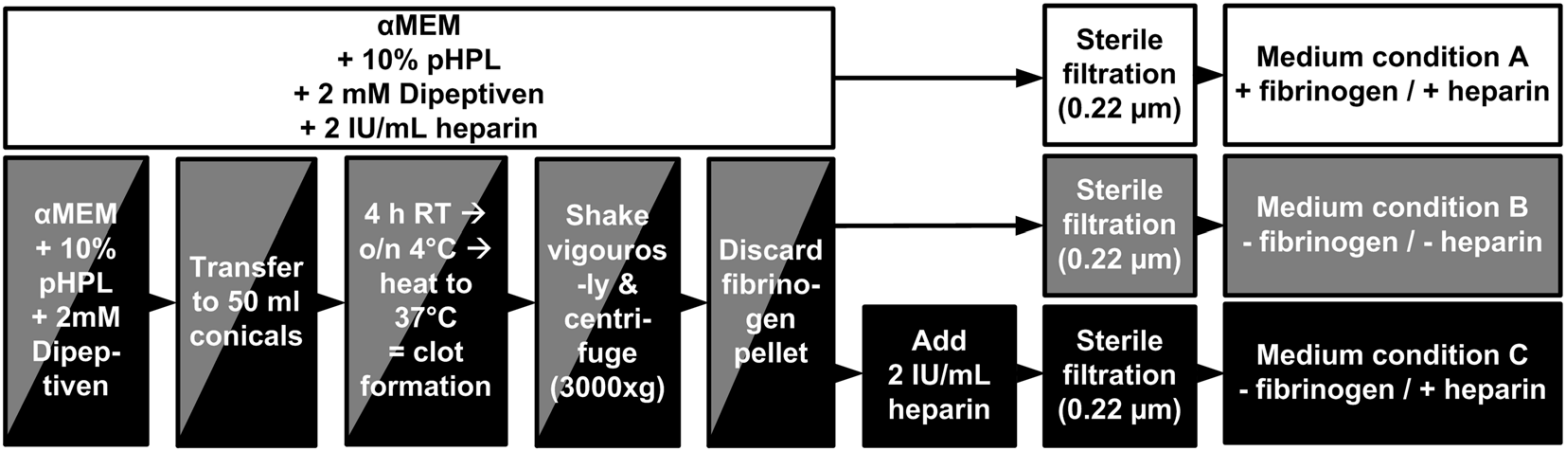
Production of standard medium (condition A), fibrinogen-depleted medium without (condition B) and with heparin (condition C). To prepare standard medium A (*white*), αMEM was supplemented with 10 % pooled human platelet lysate (pHPL), 5.5 mM Dipeptiven and 2 IU/mL heparin. Medium B (*grey*) and C (*black*) were prepared by supplementing αMEM with 10 % pHPL and 5.5 mM Dipeptiven and by allowing clot formation over night (o/n) followed by mechanical disruption of the fibrin gel by vigorous shaking. After removal of the fibrin pellet medium B was ready to use, while medium C was additionally supplemented with 2 IU/mL heparin

Three different media were prepared (medium A, B, C) using alpha-modified Minimum Essential Medium Eagle (αMEM M4526, Sigma Aldrich, St. Louis, MO, USA) supplemented with 10 % pHPL and 5.5 mM (N2)-L-Alanyl-L-Glutamin (Dipeptiven, Fresenius Kabi, Graz, Austria). pHPL was produced as described with minor modifications [18]. In brief we used outdated buffy-coat derived platelet concentrates (PCs) prepared from regular healthy blood donors at the Department for Transfusion Medicine. For each batch five PCs blood group O were pooled with five PCs blood group A or B. Platelet lysis was induced by several freeze/thaw steps (−30 °C/37 °C) and platelet fragments were depleted by centrifugation (4000×*g*, 15 min). Suitable aliquots were stored at −30 °C until use. Dipeptiven was used due to long-lasting stability in culture medium compared to standard L-Glutamin. Medium A (+fibrinogen/+heparin) was prepared as previously described [18] with 2 IU/mL of stabilisator-free porcine heparin (Biochrom, Berlin, Germany) to prevent clotting (Fig. 1, white bars) and is referred to as standard medium. Media B and C were produced by intentional hydrogel formation after pHPL supplementation in the absence of heparin. After transferring the supplemented αMEM to 50 mL conical tubes, hydrogel formation was allowed during an incubation for 4 h at room temperature (RT) followed by overnight (o/n) incubation at 4 °C. Finally the coagulated medium was heated to 37 °C (1 h) to allow complete fibrin clotting. The collapse after hydrogel formation was induced by vigorous shaking of the conical tubes followed by a centrifugation step (10 min at 3000×*g*, RT). The resulting clear medium supernatant was filtered through a 0.22 µm filter (Merck Millipore, Billerica, MA, USA). While medium B (−fibrinogen/−heparin, Fig. 1, grey) remained without further supplement, medium C (−fibrinogen/+heparin, Fig. 1, black) was supplemented with 2 IU/mL of heparin. After fibrinogen depletion, the volume recovery of media B and C was measured. The concentration of residual fibrinogen was determined in quadruplicate for three independent medium preparations by ELISA (Abcam, Cambridge, UK) according to the manufacturer’s instructions.

### Experimental setup, isolation and propagation of MSCs

The study was performed in accordance with the Helsinki Declaration. All donors signed an informed consent concerning the research use of the donated whole blood for pHPL production and of donated umbilical cord (UC) or bone marrow (BM) tissue. UC-MSCs (n = 10) and BM-MSCs (n = 3) were isolated as described previously [16, 34, 35]. Antibiotics (100 mg/mL streptomycin and 62.5 mg/mL penicillin, LifeTechnologies, Carlsbad, CA, USA), were used for initial isolation of UC-MSCs only and removed after the first 48 h. All subsequent culture conditions lacked antibiotics and cells were cultured at 37 °C, 5 % CO_2_ and 95 % humidity.

**Fig. 2:**
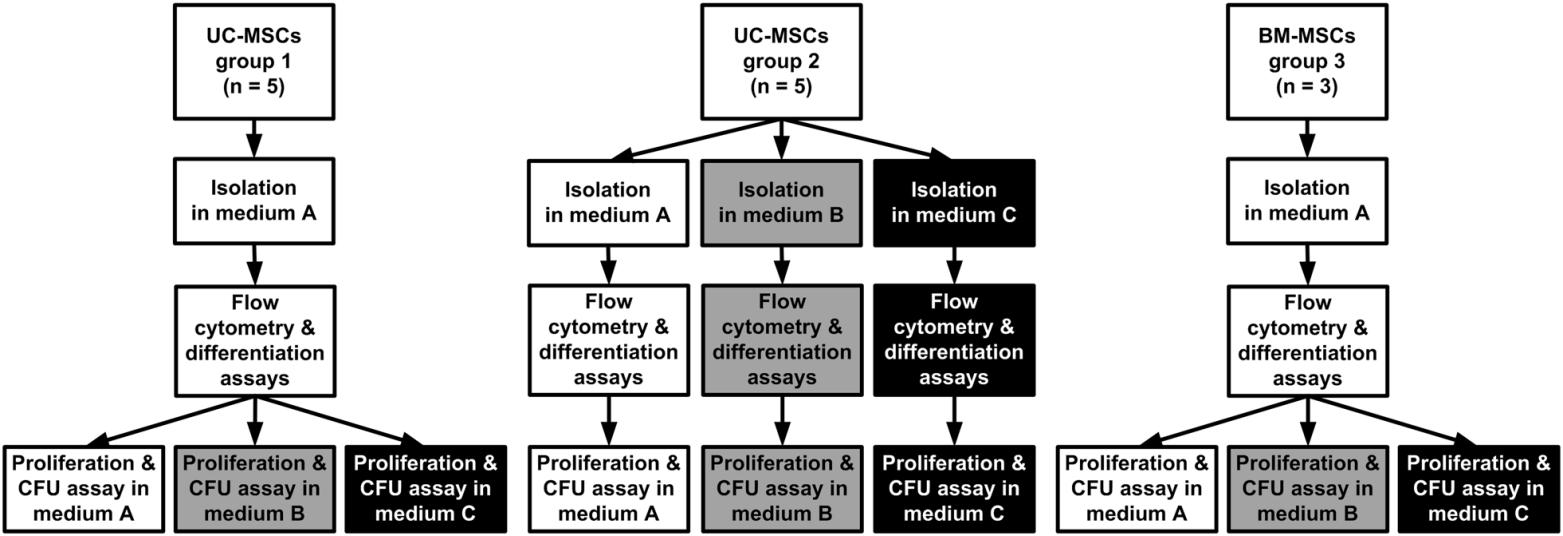
Experimental workflow: Isolation and characterization of umbilical cord (UC-) and bone marrow (BM-) MSCs. UC-MSCs group 1 (*left*) and BM-MSCs, group3 (*right*) were isolated using medium condition A exclusively. UC-MSCs group 2 (*middle*) were isolated in all three medium conditions (A, *white*; B, *grey* and C, *black*). After isolation and expansion, all MSC types tested by flow cytometric analysis and differentiation assays. Proliferation and colony forming unit assays (CFU) were performed independently in triplicates over a minimum of seven population doublings (UC-MSCs: passages 1–3, BM-MSCs: passages 2–4) examining all three medium types (A, B and C)

UC-MSCs of five independent donations (group 1, Fig. 2) were initially isolated using standard medium A only. Another five donations (group 2) were immediately separated into three cord pieces and differentially isolated in either medium condition A, B or C. We further compared the functional response of UC-MSCs to various media types with BM-MSCs (group 3). Because we regularly collected BM-aspirates using heparin, we took BM-MSCs formerly isolated in standard medium A (corresponding to UC-MSCs group1) as controls. After expansion, all MSCs were characterized by flow cytometric analysis and differentiation assays as well as by proliferation and colony forming unit (CFU) assays over three subsequent passages (Fig. 2).

### Flow cytometric analysis

MSCs were incubated in blocking buffer (5 % sheep serum (Eubio, Vienna, Austria) in PBS) in the dark at 4 °C for 25 min. Cells were stained with fluorescein isothyocyanate (FITC), phycoerythrin (PE) or allophycocyanin (APC)-conjugated mouse anti-human antibodies and corresponding isotype controls (Table 1) in flow cytometry buffer (PBS and 2 % FBS) at 4 °C in the dark for 25 min. Cells were washed, and analyzed using a FC500 flow cytometer (Beckman Coulter, Brea, CA, USA). A minimum of 10,000 nucleated cells was acquired and data were analyzed with FlowJo software (Vers. 7.6; TreeStar Inc., Ashland, OR, USA).

**Table 1:** Antibodies used for flow cytometric analysis of isolated UC- and BM-MSCs

Antibody	Conjugate	Company	Clone
Anti HLA-DR (MHC II)	FITC	Becton Dickinson, Franklin Lakes, NJ, USA	L243
Anti-h/m/rNG2/MCSP	PE	R&D Systems, McKinley Place, MN, USA	LHM-2
CD105	APC	Caltag Laboratories, Carlsbad, CA, USA	SN6
CD14	PE-Cy7	Becton Dickinson	MdeltaP9
CD140b	PE	Becton Dickinson	28D4
CD144 (VE-Cadherin)	APC	BioLegend, San Diego, CA, USA	BV9
CD19	FITC	Becton Dickinson	SJ25C1
CD271	PE	Becton Dickinson	C40-1457
CD31	FITC	Becton Dickinson	WM59
CD34	PE	Becton Dickinson	8G12
CD45	APC	Becton Dickinson	HI30
CD73	PE	Becton Dickinson	AD2
CD90	FITC	Immunotech, Quebec, Canada	F15-42-1-5
Mouse IgG1	FITC	Becton Dickinson	X40
Mouse IgG1	PE	Becton Dickinson	X40
Mouse IgG1	APC	Becton Dickinson	X40
Mouse IgG2a	PE	Becton Dickinson	X39

### Differentiation assays

The adipogenic, osteogenic and chondrogenic differentiation potential of group 1 and group 2 UC-MSCs and BM-MSCs (group 3) was tested after expansion in the particular medium type used for the primary isolation (adipogenic differentiation: passage = 2, osteogenic and chondrogenic differentiation: passage = 3). For osteogenic and adipogenic differentiation, 1000 BM- or UC-MSCs/cm^2^ were seeded. After 24 h, medium was replaced by differentiation medium as described [36]. At day 21, cells were fixed using 4 % paraformaldehyd (PFA, Sigma Aldrich) and stained with either 0.5 % Alizarin Red (Sigma Aldrich) or 1 % Sudan III (Sigma Aldrich). Chondrogenic differentiation was induced using 250,000 cells per pellet cultivated in hMSC chondrogenic SingleQuots (Lonza, Basel, Switzerland) in the presence of TGF-β3 (20 µg/mL) for 21 days. Pellets were harvested by centrifugation (1500×*g* for 5 min), fixed in 4 % PBS buffered formalin and paraffin-embedded. After deparaffination of 2 µm sections in graded alcohols, 1 % Alcian Blue staining solution (8GS, Gatt-Koller, Absam, Austria) and Nuclear Fast Red solution (Sigma Aldrich) was applied for 15 min (Multistainer platform, Leica, Wetzlar, Germany). Photographs were taken using a PrimoVert Light microscope equipped with an AxioCam ERc5 s digital camera (both from Zeiss, Oberkochen, Germany).

### Proliferation and colony forming unit (CFU) assays

To test proliferation, 1300 UC-MSCs/cm^2^ and 3300 BM-MSCs/cm^2^ were seeded in all three medium types. After 96 h, cell number was determined using “Neubauer improved” counting chambers (C-Chip, Biochrom). Cell growth was evaluated by total cell counts and cumulative population doublings (cPD) by means of the formula ln(N)/ln(2), where N is the cell number of detached cells divided by the number of cells seeded [37]. To investigate colony forming capacity, 3 MSCs/cm^2^ were seeded in cell culture dishes and cultured for 14 days. Colonies were fixed in 4 % PFA (Sigma Aldrich) and stained with 0.05 % Crystal Violet (Sigma Aldrich). Colonies were counted visually. Each assay was done in triplicate over three subsequent passages (BM-MSCs: passages 2–4, UC-MSCs: passages 1–3).

### Statistical analysis

Data are presented as arithmetic mean ± standard deviation (SD). Data were compared using 2-way ANOVA and Bonferroni multiple comparison test. Significant results are signed by asterisks (p < 0.001 or p < 0.05). Analysis was done with GraphPad Prism 5 (GraphPad Software, La Jolla, CA, USA).

## Results

### Mechanical clot depletion results in significant reduction of fibrinogen and high rates of media volume recovery

**Fig. 3:**
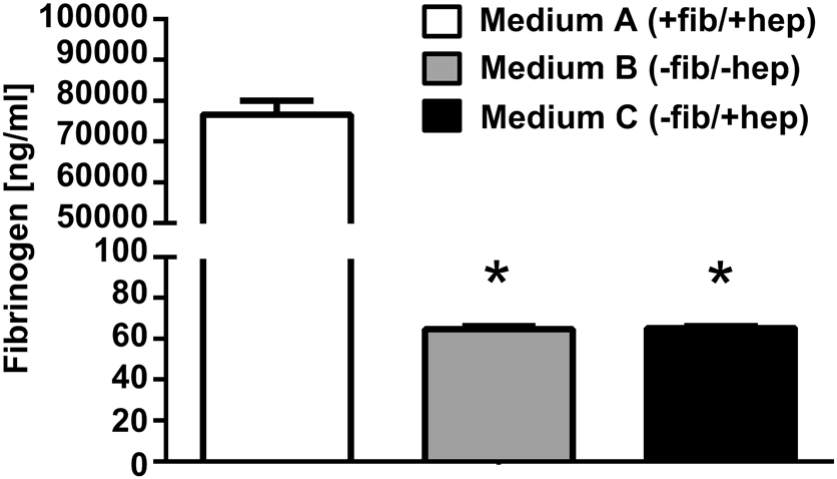
Quantitative ELISA reveals efficient depletion of fibrinogen. Quantitative ELISA demonstrated that fibrinogen concentration in depleted media B and C is 1000-fold lower compared to standard medium A. Data shown are mean values of three independent medium preparations measured in quadruplicates ± standard deviation (SD). The significance level is indicated by asterisks (* p < 0.001)

Following hydrogel formation, disruption and centrifugation, the filtration step resulted in a clear and liquid medium without delayed fibrin precipitation over 4 weeks. Measurement of fibrinogen levels in standard (medium A) compared to fibrinogen-depleted media (B and C) showed an efficient reduction from 70,935 ng/mL starting concentration to more than 1000-fold lower fibrinogen levels (66.2 and 64.5 ng/mL, respectively, Fig. 3). After clot formation and centrifugation the volume recovery was 99.5 ± 0.2 % (n = 10).

### Phenotype and in vitro functionality of human UC- and BM-MSCs are independent of fibrinogen and heparin

**Fig. 4:**
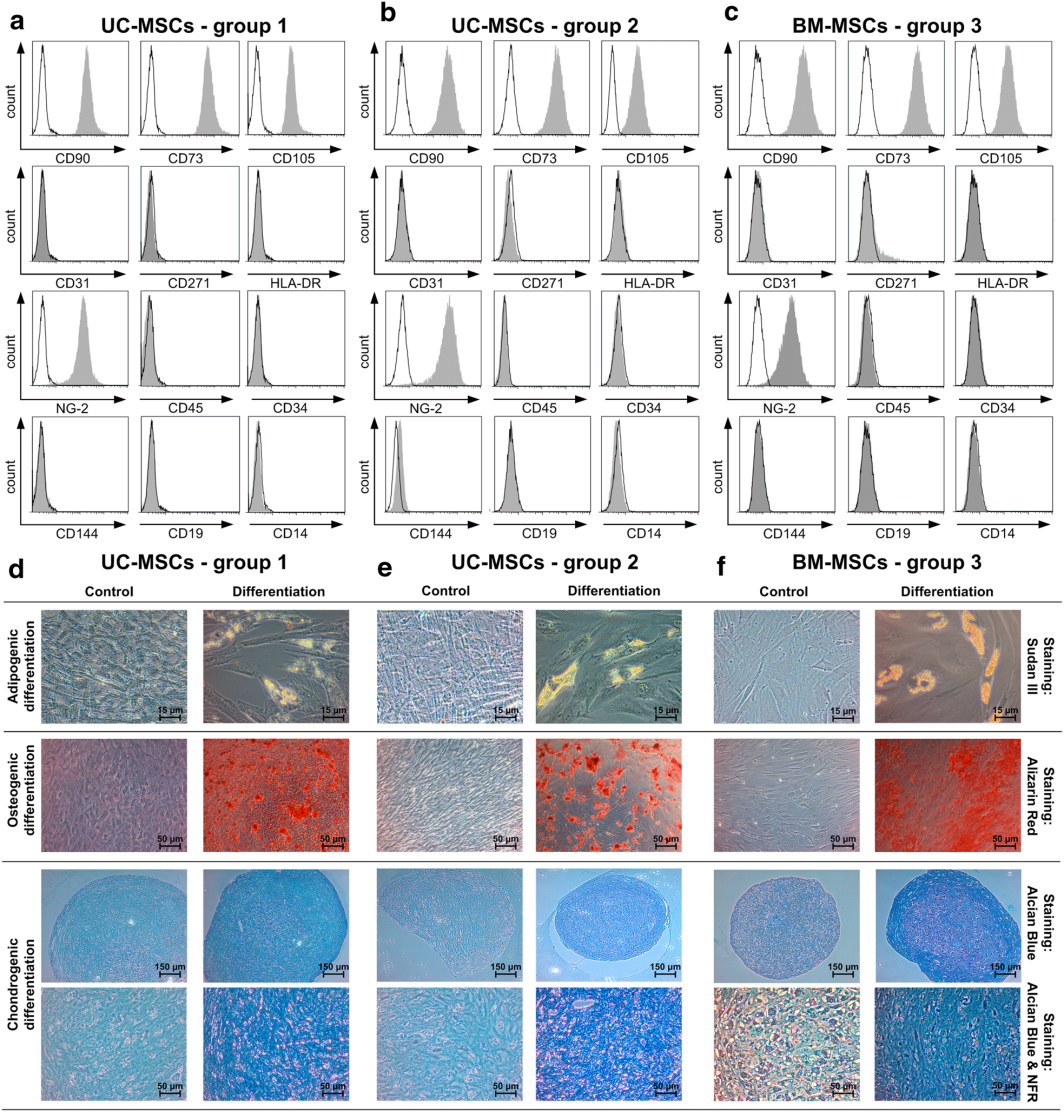
MSCs display characteristic immunophenotype and trilineage differentiation potential independent of medium type. Flow cytometric analysis of UC-MSCs, group 1 (**a**), UC-MSCs, group 2 (**b**) and BM-MSCs, group3 (**c**) revealed comparable immunophenotype profiles: CD73^**+**^/90^**+**^/105^**+**^/NG-2^**+**^ and CD14^**−**^/19^**−**^/31^**−**^/34^**−**^/45^**−**^/144^**−**^/271^**−**^/HLA-DR^**−**^. Histogram plots show representative results of UC-MSCs, group 1 consistently propagated in medium A (+fib/+hep), UC-MSCs, group 2 in medium C (−fib/+hep) and BM-MSCs, group 3 in medium A. **d**–**f** UC- and BM-MSCs display a similar in vitro trilineage potential for adipogenic (Sudan III staining), osteogenic (Alizarin *Red* staining) and chondrogenic (Alcian *Blue* staining, Nuclear *Fast Red* (NFR) staining) differentiation compared to controls. Total magnification: 200× adipogenic differentiation, 100× osteogenic differentiation, 40× and 100× chondrogenic differentiation

Flow cytometry of UC-MSCs initially isolated in standard medium (group 1), of differentially isolated UC-MSCs (group 2) and of BM-MSCs (group 3) revealed a consistent surface protein expression pattern (CD73^+^/90^+^/105^+^/NG-2^+^ and CD14^−^/19^−^/31^−^/34^−^/45^−^/144^−^/271^−^/HLA-DR^−^) characteristic for MSCs (Fig. 4a–c). Discrete variations in surface marker expression were attributed to donor variability, but were independent of medium conditions during isolation and propagation (Additional file 2: Figure S2). Tri-lineage differentiation potential evaluation showed no major differences between cell and medium types. Following adipogenic induction, BM-MSCs showed more and larger lipid droplets compared to both UC-MSC groups (Fig. 4d–f, upper panel). All MSCs showed comparable chondrogenic differentiation potential as demonstrated by Alcian Blue staining (Fig. 4d–f, lower panel). No variations in differentiation potential were observed within UC-MSC group 2: independent of medium condition A, B or C UC-MSCs demonstrated comparable tri-lineage differentiation potential (data not shown).

### Heparin increases proliferation of UC- MSCs in the absence of fibrinogen

**Fig. 5:**
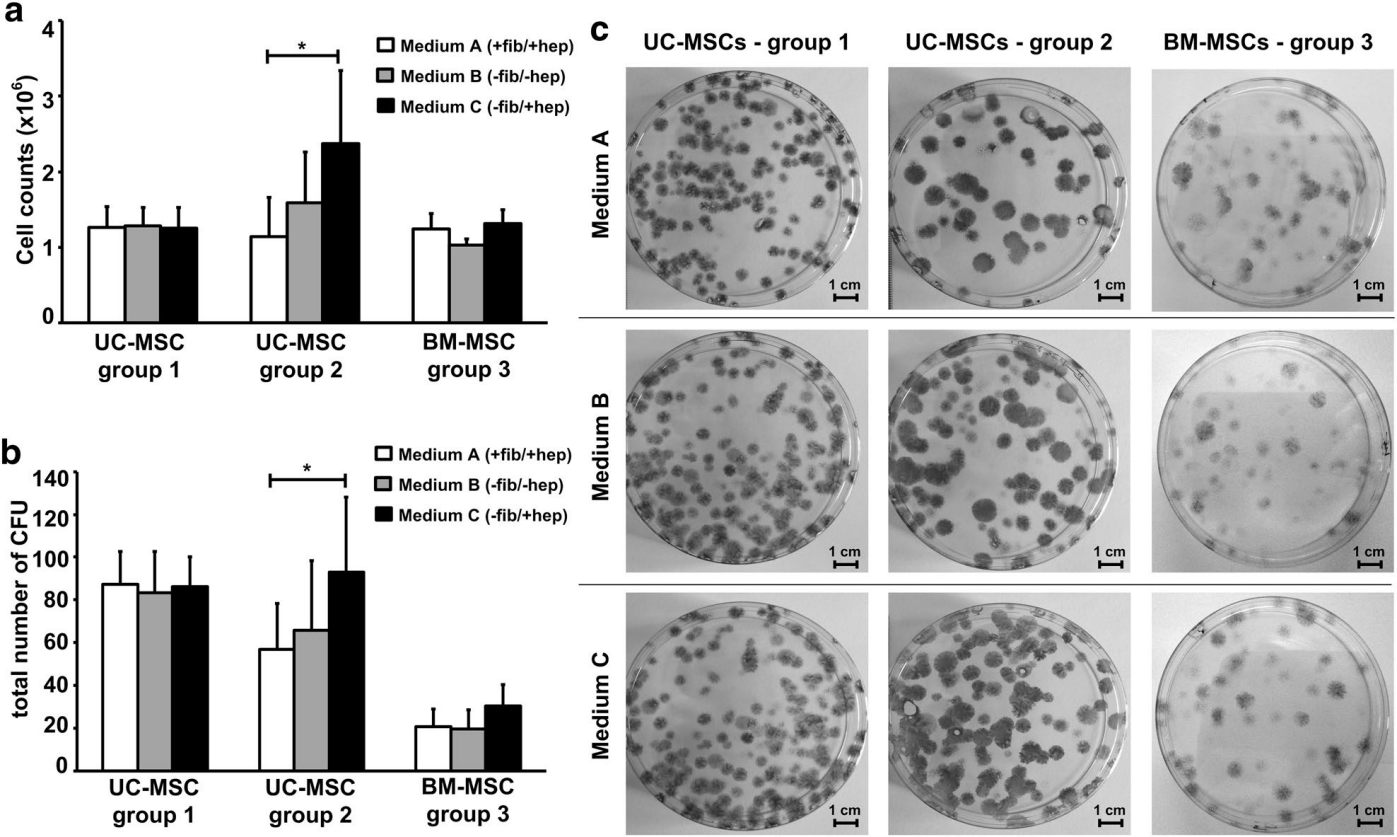
Proliferation and colony forming capacity of UC- and BM-derived MSCs. **a** The proliferative capacity of UC-MSCs, group 1 (*left*) and of BM-MSCs, group 3 (*right*) is comparable in the presence and absence of fibrinogen and heparin. UC-MSCs, group 2 (*middle*) show significantly enhanced mitogenic response to heparin if consistently isolated and cultivated in presence of heparin in fibrinogen-depleted medium (medium C) compared to standard medium A (+fib/+hep). All data shown are mean values of total cell counts of three subsequent passages done in triplicates ± standard deviation (SD) of five (UC-MSCs, group 1 and group 2) or three (BM-MSCs, group 3) independent donors. **b** UC-MSCs, group 1 (*left*) show equal colony forming capacity independent of the medium type used. Number of CFUs in UC-MSCs, group 2 (*middle*) is comparable between medium A (+fib/+hep) and B (−fib/−hep), but significantly enhanced in the presence of heparin (medium C; −fib/+ hep). Colony count of BM-MSCs, group 3 was reduced in comparison to UC-MSCs but independent of media conditions. Data shown are mean values of total colony counts of three subsequent passages done in triplicates ± standard deviation (SD). The significance level is indicated by asterisks (* p < 0.05). **c** UC-MSCs, group 1 showed smaller and more colony forming units compared to UC-MSCs, group 2 and BM-MSCs, group 3. CFU numbers of UC-MSC group 1 and BM-MSCs were independent of the medium type used for MSC isolation and cultivation. UC-MSC group 2 showed significantly more colonies in medium C (−fib/+hep), as compared to standard medium A (+fib/+hep) and medium B (−fib/−hep). Images show representative colonies of each MSC group, all passage = 2

Proliferation assays performed with the three different medium conditions (A, B or C) revealed no significant differences between the cell counts of UC-MSCs group 1 and BM-MSCs (group 3) that had been exclusively isolated in standard medium A (Fig. 5a and Additional file 1: Figure S1). UC-MSCs (group 2) initially isolated and further propagated either in the presence (medium A) or in the absence of both, fibrinogen and heparin (medium B) showed similar proliferative activity compared to UC-MSCs group 1 and BM-MSCs (group 3). After primary isolation of UC-MSCs in medium C, the persistent presence of heparin in fibrinogen-depleted medium resulted in a sustained and pronounced proliferative response (Fig. 5a and Additional file 1: Figure S1). These results were partially mirrored in CFU assays (Fig. 5b, c). While the total cell counts reached similar levels as compared to UC-MSCs group 1 the corresponding CFU numbers were reduced in UC-MSC group 2 propagated in standard medium A (fib+/hep+). A similar, but more pronounced effect was observed for BM-MSCs: While colony number was reduced, the total cell count was comparable with UC-MSC group 1. This could be attributed to the high proliferative activity of single clones resulting in larger colony sizes (and higher cell numbers per colony). One obvious difference was the consistently weak Crystal Violet staining that was found with BM-MSCs as compared to UC-MSCs. However cell density per colony was high, thus MSC counts reached comparable levels to UC-MSC group 1 (Fig. 5c).

These data point to a significant mitogenic effect of heparin on primary UC-MSCs, when isolated and permanently cultured in the absence of fibrinogen. However, if MSCs had former contact with fibrinogen (UC-MSC group 1 and BM-MSC, group 3) this response to heparin was not observed, suggesting that the mitogenic effect of heparin might be masked by fibrinogen.

## Discussion

Human MSCs are key candidates for cell therapy with the intention to treat immune disorders like graft-versus-host disease, or multiple sclerosis, inherited tissue defects such as osteogenesis imperfecta and traumatic, toxic or ischemic organ damage [38–41]. Animal components are not recommended to be used in clinical trials and can be replaced by pHPL derived from human platelet rich plasma (PRP) [17, 18]. To avoid unintended clot formation of plasmatic coagulation factors in pHPL-supplemented medium, the addition of porcine-derived heparin is commonly used. Although porcine heparin has been clinically applied for decades, some reports have pointed out severe side effects such as thrombocytopenia, hyperkalemia and hypersensitivity in up to 5 % of the patients, which is ascribed either to the product itself, the impurities contained within the product or the type of preparation [42–48]. Attempts to produce heparin by chemoenzymatic synthesis have been reported and may help to avoid the porcine product in human cell culture [49]. However, even if synthesized successfully it would be necessary to demonstrate similar in vivo and in vitro effects of synthetic heparin on MSC functionality, such as immunomodulatory potential, stemness and proliferative, migratory as well as homing capacity [28, 30, 50, 51].

In order to avoid the addition of heparin, work from Copland et al. and Mojica-Henshaw et al. suggested the reduction of heparin and also of fibrinogen from complete cell culture growth media formulations [30, 31]. Their proposed strategy used forced gelation of platelet lysate by addition of CaCl_2_ prior to cell culture medium supplementation. This method may be limited by (1) nonphysiologic calcium levels, that in turn may negatively influence the osteogenic properties of MSCs [52], and (2) by poor volume recovery of fibrinogen-depleted platelet lysate. Only 60 % of the HPL starting volume was recovered after the clotting procedure by Copland et al. [30]. The method presented in this study allows clot formation of pHPL supplemented medium and achieves a 99.5 % medium recovery rate, while achieving a more than 1000-fold fibrinogen reduction. In contrast to protocols that suggest a minimum concentration of heparin (0.6 IU/mL) [26], our heparin-free method represents a robust procedure resulting in a clear cell culture medium without delayed further clotting or fibrin precipitation. The method can be performed in a highly standardized manner, omits the addition of nonessential reagents, facilitates an economic use of pHPL as human alternative to FBS and thus provides an efficient and reproducible preparation of complete cell culture growth medium.

pHPL as an alternative to FBS is increasingly used in the scientific community and is under investigation as an important supplement in fully humanized culture systems for the generation of cellular therapeutics. It is regarded as rather safe concerning infectious diseases due to the routinely tested infectious parameters (for most European blood centers: HIV1/2, HAV, HBV, HCV, TPHA and Parvo B19). However, there is a certain risk of transmitting viruses that are not routinely tested in blood donors. Therefore, in 2014 Castiglia et al. suggested to subject pHPL to pathogen inactivation (iHPL) by psoralen and showed that there were no significant differences between pHPL and iHPL concerning colony-forming unit number, immunophenotype or multipotent capacity of BM-MSCs [53]. However, the effect of psoralen on MSCs expanded in iHPL has not yet been investigated in more detail. Further studies are required to demonstrate the role of psoralen in cell culture of MSCs for clinical applications. In addition, there are reports about patients with allergic reactions to psoralen [54]. In order to circumvent psoralen, pathogen inactivation can also be done with riboflavin in combination with UVA or using UVC only. However, several studies show that all of the pathogen inactivating treatments influence the proteome and activation states of platelets [55, 56]. This might impact on functionality of cell therapy products generated in iHPL. As an alternative, quality tests for a broad range of contaminating viruses, which are performed on a non-routine basis, are discussed to ensure safety and purity of cell therapy products [57]. However, results of a risk-based analysis performed in our cell production facility, an academic GMP-laboratory, did not argue for extended virus testing.

Analysis of the different media formulations for proliferative and functional support of UC-MSCs and BM-MSCs revealed no significant differences in proliferation, colony forming capacity, phenotype and in vitro tri-lineage differentiation, if MSCs were isolated in the presence or absence of both, fibrinogen and heparin. In CFU assays we found higher colony numbers in UC-MSCs group 1 as compared to group 2 in standard medium, a difference which was not reflected in absolute cell counts. This effect can be assigned to donor variability with large size and enhanced cell density per colony compensating the observed difference in CFU numbers. Our findings support the assumption that fibrinogen-depletion does not result in a significant loss of various pHPL-supplied growth factors or components critical for adipo-, osteo- and chondrogenic induction. Therefore fibrinogen-depleted and heparin-free medium is suitable for the successful in vitro propagation of functional MSCs, suggesting that both substances are dispensable for the effective expansion of UC- and BM-MSCs.

However, we provide evidence that during isolation and further expansion of UC-MSCs, fibrinogen and heparin do critically influence at least proliferative capacity. UC-MSCs of the same donor primarily isolated and maintained in the absence of fibrinogen, but in the presence of heparin, showed an enhanced proliferation compared to UC-MSCs initially isolated in standard medium. This proliferative response to heparin was never found after an initial contact to fibrinogen. The enhanced proliferative effect of heparin on UC-MSCs isolated and cultivated in fibrinogen-depleted medium indicates a strong mitogenic stimulus induced by heparin, which may be masked by fibrinogen. These results are in line with data suggesting that heparin may interfere with proliferation and functional capacity of MSCs [23, 26, 28, 50]. Further studies are needed to investigate the molecular mechanism and a so far unknown interplay between fibrinogen, heparin and MSCs in vitro as well as putative effects on MSC functionality in vivo.

## Conclusion

In conclusion, we have shown that porcine heparin is dispensable for the propagation of MSCs from umbilical cord and bone marrow. We have developed a convenient, reproducible and GMP-compliant procedure to deplete pHPL-derived fibrinogen in MSC culture medium that can be efficiently used for MSC propagation in clinical trials.

## Additional files

10.1186/s12967-015-0717-4 MSC proliferation shown as cumulative population doublings (cPD). cPD have been calculated. The proliferative capacity of UC-MSCs, group 1 and of BM-MSCs, group 3 is comparable in the presence and absence of fibrinogen and heparin. In contrast, cPD of group 2 UC-MSCs show a significantly enhanced proliferation in response to heparin (group 2_C) compared to medium A and medium B. Data shown are mean values of cPD of three passages done in triplicates ± standard deviation (SD) of five (UC-MSCs) or three (BM-MSCs) independent donors.
10.1186/s12967-015-0717-4 Flow cytometric analysis of individual MSC donations. Flow cytometric analysis of all individual donations of UC-MSCs, group 1 (A), UC-MSCs, group 2 (B) and BM-MSCs, group 3 (C). All MSCs isolated show a characteristic immunophenotype: CD73^+^/90^+^/105^+^/NG-2^+^ and CD14^−^/19^−^/31^−^/34^−^/45^−^/144^−^/271^−^/HLA-DR^−^. No significant differences in surface marker expression could be observed if MSCs of the same donor were cultivated in different medium conditions (A, B or C); Minor variations in expression profiles were ascribed to donor variability.

## Abbreviations

BM: bone marrow
BM-MSCs: bone marrow-derived mesenchymal stem cells
Fib: fibrinogen
GMP: good manufacturing practice
Hep: heparin
MSCs: Mesenchymal stem cells
PC: platelet concentrate
pHPL: pooled human platelet lysate
UC: umbilical cord
UC-MSCs: umbilical cord-derived mesenchymal stem cells
