# Supplementary figures and images for: Mechanical fibrinogen-depletion supports heparin-free mesenchymal stem cell propagation in human platelet lysate
Source: J Transl Med. 2015 Nov 10;13:354. doi: 10.1186/s12967-015-0717-4 (PMC4641400; doi:10.1186/s12967-015-0717-4)

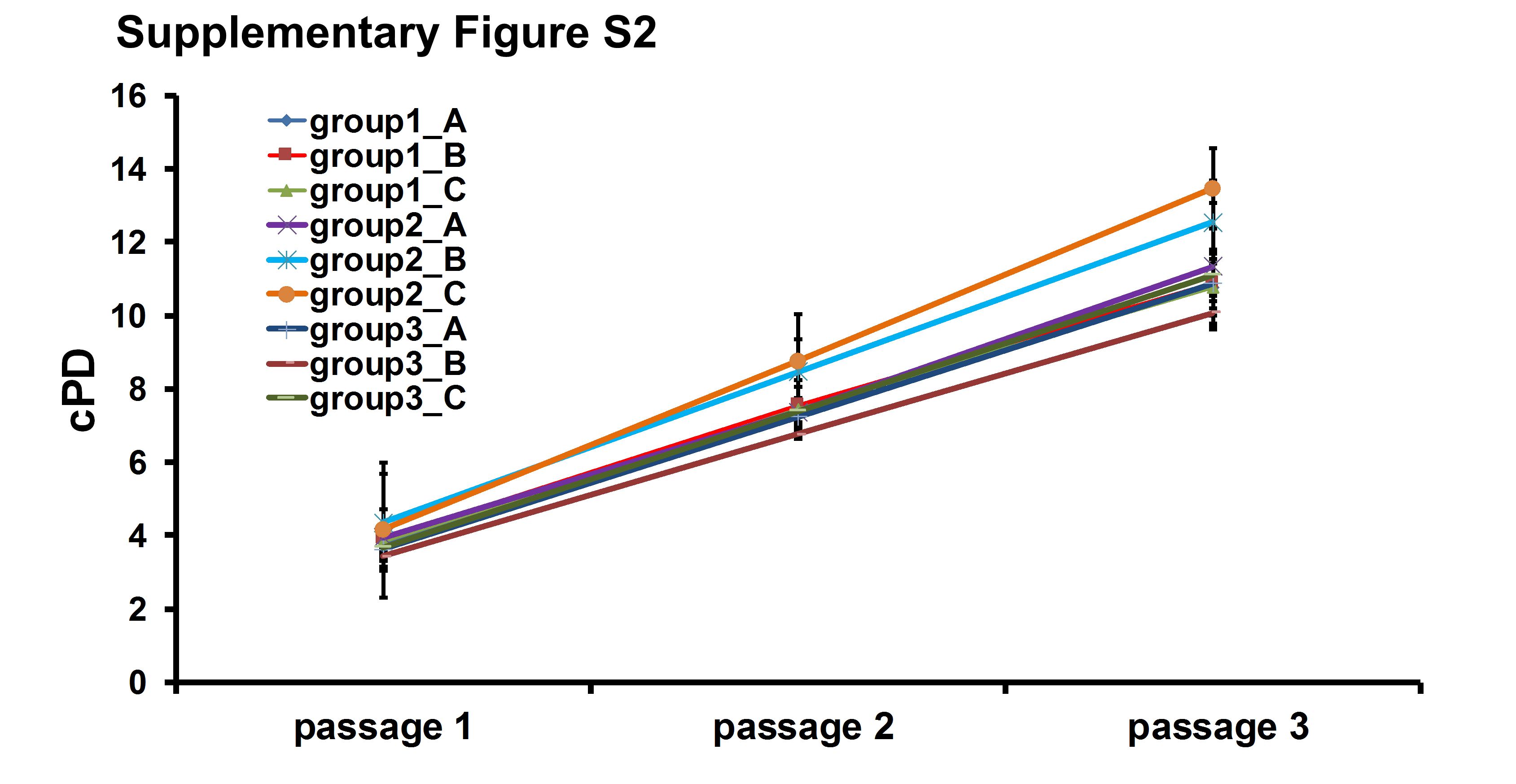

Supplement: Supplementary file 1 — 10.1186/s12967-015-0717-4 MSC proliferation shown as cumulative population doublings (cPD). cPD have been calculated. The proliferative capacity of UC-MSCs, group 1 and of BM-MSCs, group 3 is comparable in the presence and absence of fibrinogen and heparin. In contrast, cPD of group 2 UC-MSCs show a significantly enhanced proliferation in response to heparin (group 2_C) compared to medium A and medium B. Data shown are mean values of cPD of three passages done in triplicates ± standard deviation (SD) of five (UC-MSCs) or three (BM-MSCs) independent donors. [file 12967_2015_717_MOESM1_ESM.tif]

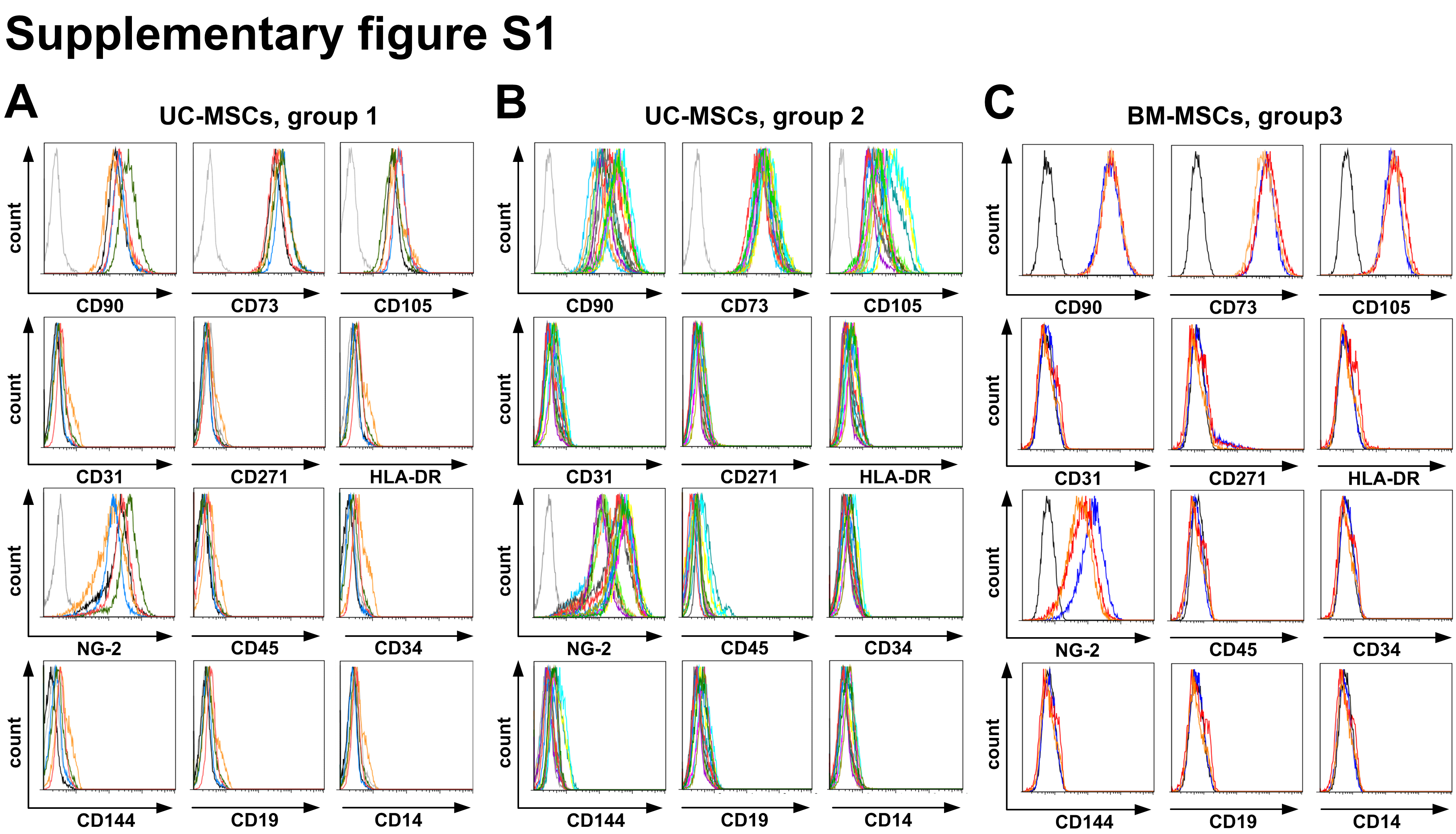

Supplement: Supplementary file 2 — 10.1186/s12967-015-0717-4 Flow cytometric analysis of individual MSC donations. Flow cytometric analysis of all individual donations of UC-MSCs, group 1 (A), UC-MSCs, group 2 (B) and BM-MSCs, group 3 (C). All MSCs isolated show a characteristic immunophenotype: CD73+/90+/105+/NG-2+ and CD14−/19−/31−/34−/45−/144−/271−/HLA-DR−. No significant differences in surface marker expression could be observed if MSCs of the same donor were cultivated in different medium conditions (A, B or C); Minor variations in expression profiles were ascribed to donor variability. [file 12967_2015_717_MOESM2_ESM.tif]
